# Comparison of Effectiveness and Safety between Intraoperative 3D-CT-Guided and C-Arm-Guided Percutaneous Balloon Compression for Idiopathic Trigeminal Neuralgia:  A  Multi-Center Retrospective Study

**DOI:** 10.1155/2021/9306532

**Published:** 2021-06-07

**Authors:** Xiong Xiao, Zhengjun Wei, Hao Ren, Hongtao Sun, Fang Luo

**Affiliations:** ^1^Department of Neurosurgery, Beijing Tiantan Hospital, Capital Medical University, Beijing 100070, China; ^2^Institute of Neurotrauma Repair, Characteristic Medical Center of the Chinese People's Armed Police Force, Tianjin 300162, China; ^3^Department of Pain Management, Beijing Tiantan Hospital, Capital Medical University, Beijing 100070, China

## Abstract

**Objectives:**

To compare 3D-CT-guided and C-arm-guided percutaneous balloon compression (PBC) in terms of effectiveness and safety.

**Methods:**

The medical records and follow-up data of patients with idiopathic trigeminal neuralgia who underwent 3D-CT-guided or C-arm-guided PBCs in Beijing Tiantan Hospital and the Characteristic Medical Center of the Chinese People's Armed Police Force between February 2018 and March 2020 were retrospectively reviewed and analysed.

**Results:**

A total of 291 patients were included. Among them, 212 patients underwent PBC treatment with 3D-CT and others with C-arm. One (0.5%) patient in 3D-CT group and 4 (5.1%) patients in C-arm group failed to receive PBC treatment because of failure of foramen ovale (FO) puncture (*P*=0.020). Among patients with successful attempts, 5 (2.4%) patients in the 3D-CT group and 11 (14.7%) patients in the C-arm group received more than one needle pass during the procedure (*P* < 0.001). The 3D-CT group required less time than the C-arm group for puncture (*P* < 0.001) and for the whole operation (*P* < 0.001). The groups shared similar initial relief rates (*P*=0.749) and similar recurrence-free survival during follow-ups for a median of 22 months (*P*=0.839). No puncture-related complications occurred in either group and the two groups had similar incidences of compression-related complications.

**Conclusion:**

3D-CT facilitated FO puncture and improved success rate of PBC. The overall time efficiency of PBC was also increased with 3D-CT. Thus, 3D-CT is a potentially useful image guidance technology for treating idiopathic trigeminal neuralgia by PBC.

## 1. Introduction

Trigeminal neuralgia (TN), with an annual incidence of 4.3–27/100,000, is one of the most common craniofacial pain syndromes [1–3]. Manifesting as paroxysmal attacks of pain that feel like electric shocks in one or more trigeminal nerve branches, TN imposes a significant burden on patients' quality of life [3]. Pharmacological interventions do not reliably bring long-lasting pain relief, and drug treatments produce severe side effects in half of all cases, leaving patients in need of surgery as an alternative [1, 4]. According to the International Classification of Headache Disorders 3rd edition (ICHD-3), TN can be classified into three subgroups: classic TN, secondary TN, and idiopathic TN (ITN) [5]. Different from other subgroups where neurovascular compression or underlying diseases can easily be identified, ITN does not show significant abnormalities in electrophysiological tests or MRI scans, which makes etiological targeted surgeries impossible [5]. Other surgeries, such as percutaneous balloon compression (PBC), gamma knife radiotherapy, glycerol rhizotomy, and radiofrequency thermocoagulation, have repeatedly been reported to be useful for pain control in TN for which pharmacological treatments have failed [1, 6–9].

Among these surgical options, PBC is a commonly used microinvasive technique and shows potential in the control of TN [6, 9, 10]. However, PBC involves the cannulation of foramen ovale (FO) which requires clinicians to overcome a challenging learning curve and is a very important procedural component [11–14]. Incorrect cannulation can hinder the success of PBC, and repeated attempts to achieve correct needle placement may cause severe complications [15]. In addition, 2–4% of patients reportedly have significant anatomic variations in the FO, making PBC an especially difficult procedure in these patients [13, 16, 17]. Clinicians are continuously searching for visual methods to help identify the puncture route and validate the positions of both the FO and the balloon to obtain more reliable puncture results and better PBC results [12, 14–16, 18]. Monoplanar and biplanar x-ray fluoroscopy was the first introduced [12, 19, 20]. In recent years, C-arm imaging has become a common assistive tool for this procedure. More advanced methods such as Dyna-CT and neuronavigation have also been reported to be helpful [11–14]. Recently, Mendes et al. reported that intraoperative CT guidance was useful in the puncture of narrow or difficult-to-access FO [17, 21]. Previous studies also reported intraoperative CT scan to be a convenient tool with advantages in operations involving FO puncture for the treatment of TN [18, 22].

However, comparison between different image-guided approaches has rarely been reported, and the application value of intraoperative CT  guidance in PBC for TN has been inadequately reported. Hence, we performed this retrospective multi-centric study to compare the effectiveness and safety of intraoperative CT-guided PBC treatment and the common C-arm-guided PBC treatment in patients with ITN.

## 2. Materials and Methods

### 2.1. Study Design and Patient Population

After obtaining approvals from the Institutional Review Boards (IRBs) of Beijing Tiantan Hospital, Capital Medical University (BTH-CMU), and Characteristic Medical Center of the Chinese People's Armed Police Force (CMC-CAPF), the medical records and follow-up data of all patients who underwent PBC for ITN in the Department of Pain Management at BTH-CMU and the Institute of Neurotrauma Repair at CMC-CAPF between February 2018 and March 2020 were retrospectively collected and reviewed for analyses. The requirement for informed consent was waived by both IRBs because of the retrospective nature of this study and de-identification was performed during data collection. This study was reported in accordance of STROBE guideline.

The inclusion criteria were as follows: (1) patients older than 18 years old; (2) patients who suffered from craniofacial pain fulfilling the characteristics of ITN as defined by ICHD-3 and who had preoperatively received a clinical diagnosis of ITN; and (3) patients who underwent intraoperative 3D-CT-guided or C-arm-guided PBC of trigeminal ganglion for the treatment of ITN. Patients whose detailed perioperative clinical data or follow-up data were incompletely recorded were excluded from this study.

### 2.2. PBC Procedures

#### 2.2.1. Preparation and Anaesthesia

C-arm is a routinely used guidance technique in PBC surgeries at both participating centers. To further improve PBC surgery, spiral-CTs were used as an alternative guidance tool. Before PBC operations, patients were explained in detail regarding the procedures of PBC surgeries with both techniques and were asked to choose which technique they preferred.

Patients were placed in supine position with their head centered on a CT scanning bed or an operating table. Blood pressure, heart rate, electrocardiography, and pulse oximetry were continuously monitored. Sufentanil (0.1 *μ*g/kg), propofol (1.5–2 mg/kg), and cisatracurium (0.1 mg/kg) were injected for anaesthesia induction. A laryngeal mask (LMA) was then applied and connected to the circle system for ventilation. Propofol (4 mg/(kg·h)) and remifentanil (0.05–0.1 *μ*g/(kg·minute)) were administered with an intravenous pump to maintain anaesthesia.

Puncture point and puncture path were infiltrated with local anaesthetic (0.5% lidocaine). Puncture was performed by an adapted Hartel approach using a 14 G needle with a semi-sharp stylet (CTZ-15, Qingyuan Medical Instrument, Shenzhen, China) through a stab incision targeting the FO.

#### 2.2.2. 3D-CT-Guided Procedure

**Figure 1:**
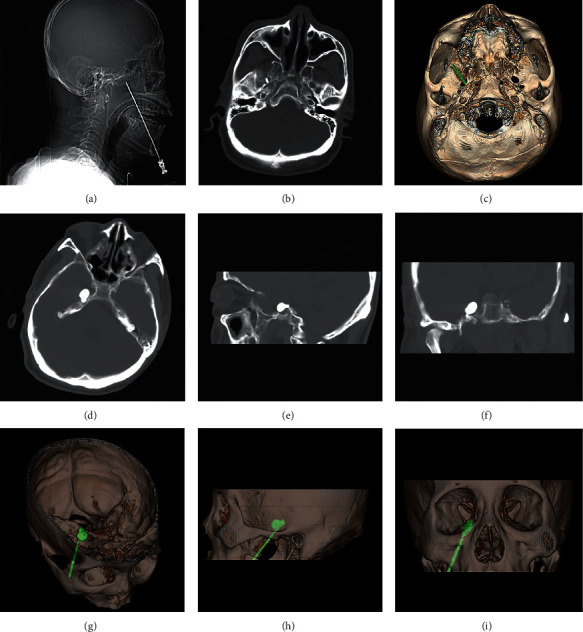
2D images and reconstructed 3D-CT  images during FO puncture. (a) Scout image of spiral CT  during FO puncture; (b), (c) 2D and reconstructed 3D images of spiral CT showing the spatial relationship between the needle and the targeted FO; (d-f) axial, sagittal, and coronal views of the pear-shaped balloon; (g-i) reconstructed 3D-CT images provided confirmation of the position and shape of the balloon.

The entry point was located approximately 2.5 cm lateral to the commissure of the lips, and the other two reference points were 3 cm anterior to the external auditory meatus along the zygomatic arch, 1 cm inferior to the pupil. Based on the surgeon's clinical experience, the needle was advanced less than 7 cm, where the tip reached near the basal part of the mid-cranial fossa. Then, CT scan was performed, and 3D reconstruction of the CT images was visualized on a post-processing workstation (GE AW VolumeShare 2, version aw4.4, Wisconsin, USA) to determine the exact location of the needle and the FO. The position of the needle was adjusted according to its spatial relationship with the FO, until it was verified by a subsequent CT scan that the needle had entered the FO (Figures 1(a)–1(c)). [22] After the stylet was withdrawn, a disposable balloon catheter (QKS-1850567, Qingyuan Medical Instrument) was inserted into the needle and then into the Meckel's cave using a guide wire. When the end of the balloon catheter was 1 cm past the end of the needle and was 17–19 mm from the FO, [8] the guide wire was removed, and 0.3–0.5 ml of nonionic contrast agent (Omnipaque) was slowly injected into the balloon catheter to inflate the balloon. Another CT scan was performed and 3D images were reconstructed to check the position and shape of the balloon (Figures 1(d)–1(i)). The balloon appears to form the shape of a pear when positioned correctly into the FO. If satisfactory position or shape was not obtained, the balloon was deflated, the catheter was withdrawn, and the needle was set back and readjusted according to the CT images, following which the balloon was reinflated, and imaging scans and evaluations were performed again [19].

#### 2.2.3. C-Arm-Guided Procedure

**Figure 2:**
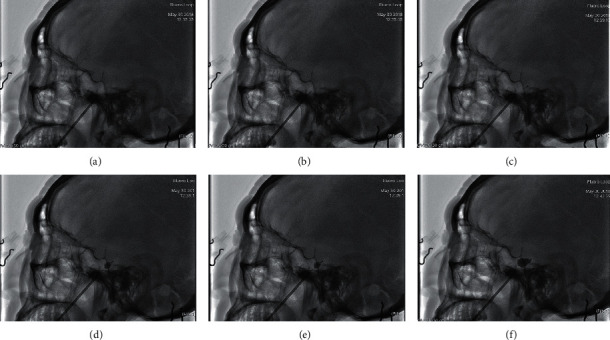
C-arm-guided FO puncture. (a) The needle penetrated the foramen ovale under lateral C-arm imaging; (b), (c) the balloon catheter was advanced into Meckel's cave under direct C-arm fluoroscopy; (d, e) the balloon was slowly inflated with nonionic contrast agent (omnipaque) under fluoroscopic monitoring; (f) the shape of the balloon was confirmed to be pear-like on lateral C-arm imaging.

C-arm image-intensified fluoroscopy was used to obtain lateral images. A needle of the same model used for the 3D-CT-guided procedure was inserted until it penetrated the FO on lateral C-arm imaging (Figure 2(a)). The same entry point and puncture direction with the CT group were used to insert the needle and identified in sagittal view as well as coronal view of the C-arm. The blunt stylet was then withdrawn, and the balloon catheter (of the same model used in the 3D-CT-guided procedure) was advanced into Meckel's cave under direct C-arm fluoroscopy (Figures 2(b) and 2(c)). The balloon was slowly inflated with nonionic contrast agent (Omnipaque) under fluoroscopic monitoring until it was proximal to the posterior fossa (Figures 2(d) and 2(e)). The shape and position of the balloon were inspected in reference to bony landmarks, such as clivus, sella turcica, and petrous bones (Figure 2(f)). If satisfactory position and pear shape was not achieved, the procedure was repeated for proper placement of the needle, by deflating the balloon, readjusting the catheter and reinflating the balloon, along with reevaluation through imaging scans.

#### 2.2.4. Procedure for Compression

After visual confirmation that the balloon was correctly positioned, a total of 0.3–0.8 ml of contrast agent was injected to compress the nerve [8]. The compression lasted 1.5–3 minutes, depending on the operator's surgical experience [8], after which the balloon was drained and removed along with the needle. The puncture point was compressed for 5 minutes to stop the bleeding, sterile dressing was then applied, and anaesthesia was stopped. After opening their eyes and having the LMA removed, patients were sent for post-anaesthesia care.

### 2.3. Data Acquisition and Analysis

We collected preoperative, intraoperative, and postoperative data from patients' medical records and the follow-up databases of both hospitals. Preoperative data included gender, age, length of history before PBC, side of TN, involved branches of the trigeminal nerve, preoperative Barrow Neurological Institute (BNI) pain score, and previous surgical treatments. Intraoperative details on the PBC procedures, such as duration of FO puncture, number of needle passes during the procedure, duration of the whole operation, and intraoperative complications and side effects, were also collected. The postoperative data included the BNI pain score, the BNI facial numbness score, and other complications or side effects.

The follow-up databases of both hospitals were built for improving the quality of medical care, and to be used for scientific research after IRB approval is granted. Each patient was clinically evaluated in person before PBC procedure and before discharge. Follow-ups were conducted at 1 month, 2 months, 3 months, and 6 months postoperatively, and then every 6 to 12 months thereafter by telephone calls or even outpatient visits if necessary. Detailed information on pain relief, pain recurrence, complications, and side effects at each follow-up was recorded in the databases.

The intensity of pain was assessed using the BNI pain intensity score [23]. Initial pain relief was defined as a BNI pain score of I or II, achieved within 3 months after PBC treatment. Pain recurrence was defined as an increase in the BNI pain intensity score from class I or II to class III or higher. Numbness was graded using the BNI facial numbness score [23]. Similar to previous studies, the number of needle passes required for successful punctures was recorded and analysed [16, 24]. A needle pass during the procedure is defined as the attempt of moving the needle forward. Before satisfactory position and shape of the balloon were achieved, if a backward movement of the needle or another attempt at FO puncture was performed for any reason, it was regarded as another needle pass.

IBM SPSS Statistics version 23 was used for statistical analyses. For measurement data, if the variables followed a normal distribution, means and standard deviations were calculated, and *t*-test or analysis of variance was used for intergroup comparisons. Otherwise, for non-normal distributions, expressed as medians and interquartile ranges (IQRs), quartiles were calculated, and Mann–Whitney U test or Kruskal–Wallis H test was used for intergroup comparisons. For categorical data, frequencies and percentages were calculated, and chi-squared test was used for intergroup comparisons. Recurrence-free survival of both groups was estimated and compared using Kaplan–Meier method.

## 3. Results

### 3.1. Patient Population and Preoperative Conditions

**Figure 3:**
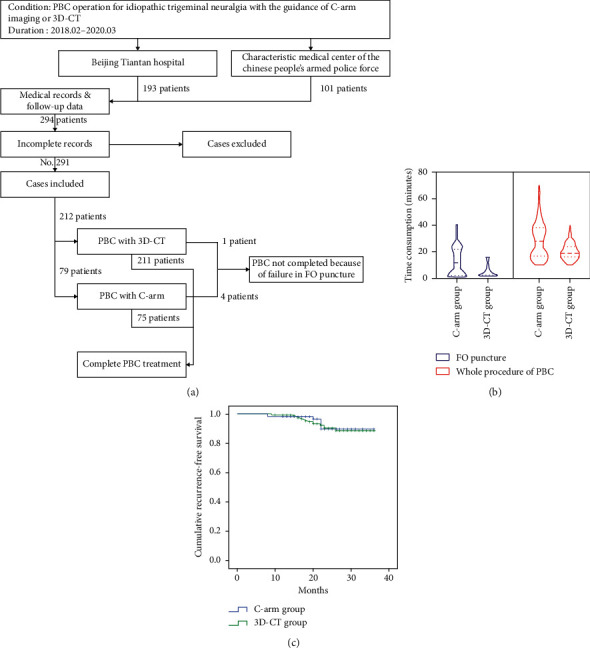
Summary of study results. (a) Plot of patient selection procedure; (b) time consumption of FO puncture and whole procedure of PBC; (c) Kaplan–Meier curves of recurrence-free survival of both groups.

A total of 294 patients were identified, among which 291 patients were included in analysis. The number of patients who underwent PBCs with C-arm and 3D-CT was 79 (27.1%) and 212 (72.9%), respectively. The patient selection procedure is shown in Figure 3(a). A summary of the patients' parameters is shown in Table 1.

### 3.2. Effectiveness of PBC Treatments

4 (5.1%) patients in the C-arm group and 1 (0.5%) patient in the 3D-CT group received an incomplete PBC procedure because of failure of FO puncture despite repeated attempts during the surgery (*P*=0.020); therefore, other therapies were sought instead. Among patients with successful attempts, 5 (2.4%) patients in the CT group and 11 (14.7%) patients in the C-arm group required more than one needle pass to achieve correct FO cannulation (*P* < 0.001). Initial pain relief was achieved in 202 (95.7%) patients in the 3D-CT group and 71 (94.7%) in the C-arm group (*P*=0.749, Table 2).

Median time taken for FO puncture was significantly shorter with the guidance of 3D-CT than with C-arm (3 minutes [IQR: 3 minutes–7 minutes] vs 12 minutes [IQR: 2 minutes–22 minutes], *P* < 0.001). In particular, the maximum time taken for FO puncture was higher in C-arm group than in 3D-CT group (40 minutes vs 16 minutes). With the guidance of 3D-CT, the median whole duration of PBC operations was also shorter (19 minutes [IQR: 16 minutes–24 minutes] vs 28 minutes [IQR: 17 minutes–38 minutes], *P* < 0.001). Additionally, the maximum time taken for the whole operation was higher in C-arm group (70 minutes VS 40 minutes). Related results are shown in Figure 3(b).

Among patients who showed initial pain relief after PBCs, the length of follow-up ranged from 8 months to 36 months, with a median of 22 months. 2 patients (2.8%) in the C-arm group and 5 patients (2.5%) in the CT  group were lost to follow-up by the time of data acquisition. Five patients (7.0%) in the C-arm group and 15 (7.4%) in the CT group suffered from pain recurrences during follow-ups. Survival analysis revealed no significant difference in recurrence-free survival between groups (*P*=0.839, Figure 3(c)).

### 3.3. Safety of PBC Treatments

No puncture-related complications were observed among all included patients. Among patients with successful attempts, common compression-related complications showed no significant difference in incidence between groups (Table 2).

## 4. Discussion

In this study, we found that both the failure rate of FO puncture and the proportion of patients who need more than one needle pass to get successful attempts were higher in the C-arm group than in the 3D-CT group. Also, the C-arm group needed more time for FO puncture and the whole PBC operation. However, once the FO was punctured successfully and the PBC was performed, the two groups shared similar rates of initial pain relief, similar recurrence-free survival during follow-ups, and similar incidences of postoperative compression-related complications. Upon these findings, we conclude that 3D-CT can facilitate FO puncture and increase the success rate and time efficiency of the procedure compared to the common image-guided method, C-arm.

Time consumption of FO punctures and the entire PBC surgeries was significantly higher in the C-arm group. The increased efficiency might be attributable to the 3D visualizations reconstructed from the thin-of images acquired by CT scans. They provide intuitive, clear and accurate observations of the spatial relationship between the needle and the targeted FO. Quantitative distances and angles can also be measured. None of these benefits can be obtained through the 2D images provided by C-arm imaging, which involves repeated adjustment of the scanning arm and multi-angle 2D scans. Consequently, physicians were able to adjust the needle swiftly and accurately on the CT group, thereby saving time. Furthermore, the length of the procedure was shorter than the values reported in previous studies with other guidance techniques [13, 24]. These previous findings further support the time efficiency of 3D-CT as an image guiding tool for PBC.

In this study, we did not compare 3D-CT with other 3D visual methods, such as Dyna-CT and neuronavigation, because the devices for these imaging techniques were not available in either of the participating centers. Although 3D-CT can provide only semi-real-time guidance, the high success rate of FO puncture was significantly different from the rates in previous reports, which stated that only 18% of neurosurgeons in training felt they would be able to perform the FO cannulation independently and FO cannulation with x-ray imaging alone could have a failure rate as high as 15%, indicating that correction of the needle after a CT scan facilitated FO puncture [12, 25]. The results concerning puncture were in accordance with previous studies on spiral CT-assisted radiofrequency treatment for TN [18, 22]. Thus, it is our opinion that 3D-CT scans are very helpful in validating and correcting needle position during PBC, making them a useful guiding technology for PBC. Meanwhile, as the C-arm is now a common tool to facilitate the FO procedure and 3D-CT showed better assistive performance, we believe that, with the assistance of 3D-CT, FO puncture is easier, and PBCs may show more potential in clinical practice. Recently, 3D C-arm, which could provide both 3D images and real-time guidance, was reported to be used in spinal operations and showed its potential [26]. However, 3D C-arm was not a common tool yet and rarely reported in TN operations. The potential of 3D C-arm will be investigated in the future if the devices are available in our sites.

We observed similar rate of initial pain relief and recurrence-free survival between the two groups. Additionally, our results were consistent with other studies concluding that the PBC method inevitably carries a high risk of facial numbness and masseter weakness [15, 27, 28]. Some researchers believe that reduced compression time can decrease the incidence of complications; however, insufficient compression time could result in unrelieved pain or pain recurrence [11]. To date, there is no consensus on the proper duration of compression or the proper volume of balloon inflation for PBC operations [24, 29, 30]. Given the retrospective nature of this study, there were no pre-set standards for compression time, and the length of compression was determined by the physicians according to their clinical experience. Furthermore, the compression settings could not be completely extracted from medical records, making it impossible to analyse whether similar safety and effectiveness was the result of imbalanced distribution of these settings among groups. Prospective controlled studies aiming at investigating the influences of both different settings and image-guided techniques are in progress.

Except PBC, other microinvasive approaches, such as gamma knife radiotherapy, glycerol rhizotomy, and radiofrequency thermocoagulation, were also reported to be useful in treatment of ITN [1, 6–9]. A recently published meta-analysis concerning TN showed that glycerol rhizotomy showed similar rate of immediate pain relief and pain recurrence with PBC but showed lower incidences of numbness and diplopia, while the incidences of other complications were similar in both groups [9]. Also, PBC showed similar rate of immediate pain relief, pain recurrence, and incidences of complications with radiofrequency thermocoagulation [9]. The reports on comparison between radiotherapy and PBC in the condition of ITN were lacking; however the reported time to pain relief was considered longer than percutaneous approaches [31]. Thus, these approaches are considered to be an option for ITN with their own potential.

This research has some limitations. First, this was a retrospective study performed in only two centers. Due to the retrospective nature of this study, patients were not assigned randomly; therefore the results may have been biased. Second, the duration of follow-up was relatively short compared to that of other published studies. Third, some valuable variables, such as the pressure of the balloon, were unavailable due to the retrospective nature of this study. To address these problems, prospective randomized studies on the effectiveness and safety of PBC with a variety of parameters for the treatment of TN must be performed in the future. In the meantime, the results of a longer follow-up of this study will be published in the next few years.

## 5. Conclusions

3D-CT facilitates FO puncture and improves the success rate of PBC procedures. The overall time efficiency of PBC is also increased with 3D-CT. Thus, 3D-CT is an image guidance technology with potential for PBCs on ITN.

## Figures and Tables

**Table 1:** Demographic data and preoperative conditions of the included patients.

Variables	Total	C-arm	3D-CT	*P* value
*N*	291	79	212	
Age (years old)	63.0 (53.9, 68.0)	63.0 (56.3, 66.9)	62.4 (53.7, 69.1)	0.882
Gender (female/male)	182/109	55/24	127/85	0.136
Preoperative disease duration (months)	47.8 ± 18.5	50.2 ± 14.6	46.9 ± 19.7	0.170
Left/right-sided lesion (n)	77/214	15/64	62/150	0.100

*Branches affected (n)*				0.237
I	15	5	10	
II	39	15	24	
III	36	10	26	
I + II	55	17	38	
I + III	19	6	13	
II + III	77	19	58	
I + II + III	50	7	43	

*Preoperative* BNI *(n)*				0.270
I	0	0	0	
II	0	0	0	
III	32	12	20	
IV	199	49	150	
V	60	18	42	
Patients with previous surgeries/interventions (n)	113	26	87	0.225
Radiotherapy	71	17	54	—
Radiofrequency	67	26	41	—
PBC	14	3	11	

**Table 2:** Summary of immediate effectiveness and postoperative complications.

	C-arm (*N* = 75)	3D-CT (*N* = 211)	*P* value
Immediate pain relief (*n*)	71	202	0.749

*Postoperative* BNI *pain score (n)*			0.266
I	43	141	
II	28	61	
III	0	1	
IV	3	8	
V	1	0	

*Complications (n)*			
Facial numbness	55	162	0.535
Hypoaesthesia	45	144	0.204
Masseter weakness	14	29	0.347
Herpes	21	65	0.770
Paraesthesia	8	15	0.330
Dysaesthesia	6	15	0.799
Diplopia	3	3	0.187
Keratitis	4	6	0.296

*Postoperative* BNI *facial numbness score (n)*			0.853
I	20	49	
II	12	36	
III	27	86	
IV	16	40	

## Data Availability

The data used to support the findings of this study are available from the corresponding author upon request via e-mail.

## References

[1] Nicola Montano N., Conforti G., Di Bonaventura R., Meglio M., Fernandez E., Papacci F. (2015). Advances in diagnosis and treatment of trigeminal neuralgia. *Therapeutics and Clinical Risk Management*.

[2] Puri N., Rathore A., Dharmdeep G. (2018). A clinical study on comparative evaluation of the effectiveness of carbamazepine and combination of carbamazepine with baclofen or capsaicin in the management of trigeminal neuralgia. *Nigerian Journal of Surgery*.

[3] Maarbjerg S., Di Stefano G., Bendtsen L., Cruccu G. (2017). Trigeminal neuralgia—diagnosis and treatment. *Cephalalgia*.

[4] Pollock B. E., Stein K. J. (2010). Surgical management of trigeminal neuralgia patients with recurrent or persistent pain despite three or more prior operations. *World Neurosurgery*.

[5] Headache Classification Committee of the International Headache Society (IHS) (2018). Headache classification committee of the international headache society (IHS) the international classification of headache disorders, 3rd edition. *Cephalalgia*.

[6] Sterman-Neto H., Fukuda C. Y., Duarte K. P. (2021). Balloon compression vs radiofrequency for primary trigeminal neuralgia: a randomized, controlled trial. *Pain*.

[7] Noorani I., Lodge A., Vajramani G., Sparrow O. (2019). The effectiveness of percutaneous balloon compression, thermocoagulation, and glycerol rhizolysis for trigeminal neuralgia in multiple sclerosis. *Neurosurgery*.

[8] Asplund P., Blomstedt P., Bergenheim A. T. (2016). Percutaneous balloon compression vs percutaneous retrogasserian glycerol rhizotomy for the primary treatment of trigeminal neuralgia. *Neurosurgery*.

[9] Texakalidis P., Xenos D., Tora M. S., Wetzel J. S., Boulis N. M. (2019). Comparative safety and efficacy of percutaneous approaches for the treatment of trigeminal neuralgia: a systematic review and meta-analysis. *Clinical Neurology and Neurosurgery*.

[10] Ni H., Wang Y., Chen X., Gu W. (2020). Outcomes of treatment for elderly patients with trigeminal neuralgia: percutaneous balloon compression versus microvascular decompression. *Journal of Craniofacial Surgery*.

[11] Huo X., Sun X., Zhang Z., Guo W., Guan N., Luo J. (2014). Dyna-CT-assisted percutaneous microballoon compression for trigeminal neuralgia. *Journal of NeuroInterventional Surgery*.

[12] Wiggins A., Lonie M., Pimentil I., Newall N., Bodkin P., Venkatesh A. (2018). Electromagnetic neuronavigation for the percutaneous treatment of trigeminal neuralgia with balloon compression: technical note and cadaveric validation study. *Acta Neurochirurgica*.

[13] Aydoseli A., Akcakaya M. O., Aras Y. (2015). Neuronavigation-assisted percutaneous balloon compression for the treatment of trigeminal neuralgia: the technique and short-term clinical results. *British Journal of Neurosurgery*.

[14] Xiaochuan H., Xiaoyun S., Junsheng L., Ning G., Wenshi G., Zhenxing Z. (2013). Percutaneous microballoon compression for trigeminal neuralgia using Dyna-CT. *Interventional Neuroradiology*.

[15] Abdennebi B., Mahfouf L., Nedjahi T. (1997). Long-term results of percutaneous compression of the gasserian ganglion in trigeminal neuralgia (series of 200 patients). *Stereotactic and Functional Neurosurgery*.

[16] Bohnstedt B. N., Tubbs R. S., Cohen-Gadol A. A. (2012). The use of intraoperative navigation for percutaneous procedures at the skull base including a difficult-to-access foramen ovale. *Neurosurgery*.

[17] Mendes P. D., Martins da Cunha P. H., Monteiro K. D. K. O., Quites L. V., Fonseca Filho G. D. A. (2021). Percutaneous foramen ovale puncture: usefulness of intraoperative CT control, in the eventuality of a narrow foramen. *Stereotactic and Functional Neurosurgery*.

[18] Lan M., Zipu J., Ying S., Hao R., Fang L. (2018). Efficacy and safety of CT-guided percutaneous pulsed radiofrequency treatment of the gasserian ganglion in patients with medically intractable idiopathic trigeminal neuralgia. *Journal of Pain Research*.

[19] Bergenheim A. T., Asplund P., Linderoth B. (2013). Percutaneous retrogasserian balloon compression for trigeminal neuralgia: review of critical technical details and outcomes. *World Neurosurgery*.

[20] Mandat T., Brozyna B., Krzymanski G., Podgorski J. K. (2009). An image-guided, noninvasive method of cannulation of the foramen ovale for awake, percutaneous radiofrequency rhizotomy. *Journal of Neurosurgery*.

[21] Guo Z., Wu B., Du C., Cheng M., Tian Y. (2016). Stereotactic approach combined with 3 D CT reconstruction for difficult-to-access foramen ovale on radiofrequency thermocoagulation of the gasserian ganglion for trigeminal neuralgia. *Pain Medicine*.

[22] Fang L., Ying S., Tao W., Lan M., Xiaotong Y., Nan J. (2014). 3 D CT-guided pulsed radiofrequency treatment for trigeminal neuralgia. *Pain Practice*.

[23] Rogers C. L., Shetter A. G., Fiedler J. A., Smith K. A., Han P. P., Speiser B. L. (2000). Gamma knife radiosurgery for trigeminal neuralgia: the initial experience of the barrow neurological institute. *International Journal of Radiation Oncology Biology Physics*.

[24] Lin M. H.-C., Lee M.-H., Wang T.-C. (2011). Foramen ovale cannulation guided by intra-operative computed tomography with integrated neuronavigation for the treatment of trigeminal neuralgia. *Acta Neurochirurgica*.

[25] Håkanson S. (1981). Trigeminal neuralgia treated by the injection of glycerol into the trigeminal cistern. *Neurosurgery*.

[26] Banat M., Wach J., Salemdawod A., Bahna M., Scorzin J., Vatter H. (2021). The role of intraoperative image guidance systems (three-dimensional C-arm versus O-arm) in spinal surgery: results of a single-center study. *World Neurosurgery*.

[27] Campos W. K., Linhares M. N. (2011). A prospective study of 39 patients with trigeminal neuralgia treated with percutaneous balloon compression. *Arquivos de Neuro-Psiquiatria*.

[28] Montano N., Ioannoni E., Rapisarda A. (2020). The risk of mastication weakness after percutaneous balloon compression for the treatment of trigeminal neuralgia. *Clinical Neurology and Neurosurgery*.

[29] Lee S. T., Chen J. F. (2003). Percutaneous trigeminal ganglion balloon compression for treatment of trigeminal neuralgia, part II: results related to compression duration. *Surgical Neurology International*.

[30] Ying X., Wang H., Deng S., Chen Y., Zhang J., Yu W. (2017). Long-term outcome of percutaneous balloon compression for trigeminal neuralgia patients elder than 80 years. *Medicine*.

[31] Tuleasca C., Régis J., Sahgal A. (2018). Stereotactic radiosurgery for trigeminal neuralgia: a systematic review. *Journal of Neurosurgery*.

